# Multilevel analysis of COVID-19 vaccination intention: the moderating role of economic and cultural country characteristics

**DOI:** 10.1093/eurpub/ckae022

**Published:** 2024-02-20

**Authors:** Monika Lamot, Andrej Kirbiš

**Affiliations:** Department of Sociology, Faculty of Arts, University of Maribor, Maribor, Slovenia; Department of Sociology, Faculty of Arts, University of Maribor, Maribor, Slovenia

## Abstract

**Background:**

Predictors of COVID-19 (coronavirus) vaccination have been extensively researched; however, the contextual factors contributing to understanding vaccination intention remain largely unexplored. The present study aimed to investigate the moderating role of economic development (Gross domestic product - GDP *per capita*), economic inequality (Gini index), the perceived corruption index and Hofstede’s measurements of cultural values—index of individualism/collectivism and power distance index—in the relationship between determinants of satisfaction with the healthcare system, trust in political institutions, conspiracy beliefs and COVID-19 vaccination intention.

**Methods:**

A multilevel modelling approach was employed on a sample of approximately 51 000 individuals nested within 26 countries. Data were drawn from the European Social Survey Round 10. The model examined the effect of individual- and country-level predictors and their interaction on vaccination intention.

**Results:**

Satisfaction with the healthcare system had a stronger positive effect on intention to get vaccinated in countries with lower perceived corruption and more individualistic countries. Trust in political institutions had a stronger positive effect on vaccination intention in countries with higher economic development and lower perceived corruption, while a negative effect of conspiracy beliefs on vaccination intention was stronger in countries with lower economic development, higher perceived corruption and a more collectivistic cultural orientation.

**Conclusion:**

Our findings highlight the importance of considering individual and contextual factors when addressing vaccination intention.

## Introduction

Prior research shows that belief in conspiracy theories negatively impacts vaccine attitudes,^1^ while trust in political^2^ and health institutions^3^ boosts it. These findings, primarily from single-country samples, have not been widely examined cross-nationally.^4^ Thus, it remains unknown whether country-level contexts affect the determinants of vaccine hesitancy. In the present study, we examine the potential moderating role of macro characteristics on the impact of determinants of COVID-19 vaccination intention.

### Economic development and inequality

Economic development has been linked to expressing belief in conspiracy theories. Evidence suggests conspiracy theories tend to proliferate in adverse economic circumstances.^5^ Drochon^6^ similarly found that conspiracy theories are more prevalent in countries with lower GDP but also in countries with higher economic inequality,^7^ as measured by the Gini index—a statistical measure of income inequality where a higher value indicates greater inequality. Higher economic inequality is also associated with lower public trust in government, fellow citizens and science.^8^ However, there is scarce research on macro characteristics as potential moderators of micro-level determinants of vaccination.^9^ Given that individuals are nested in different social contexts with complex interactions between the individual and the environment,^10^ it is vital to examine how countries’ characteristics might influence these relationships.

We hypothesize that low GDP *per capita* and a high Gini coefficient moderate the relationship between conspiracy theories, distrust in political institutions, dissatisfaction with the healthcare system (HCS), and vaccination intention, such that the effects are stronger in less economically developed and more economically unequal countries. The rationale behind this hypothesis stems from the idea that in such economically insecure societies, individuals may feel a heightened sense of vulnerability and uncertainty,^11^ making them more susceptible to conspiracy theories and more likely to distrust political institutions. Such countries may also be characterized by less robust HCSs.^12^ In essence, higher GDP is associated with better healthcare infrastructure, education, and the public’s overall well-being,^13^ which can act as buffers against the deleterious effects of negative attitudes.

*Hypothesis 1*: GDP *per capita* moderates the effect of satisfaction with the HCS (H1a) trust in political institutions (H1b) and conspiracy theory beliefs (H1c) on vaccination intention. The positive effects of trust in political institutions and satisfaction with the HCS are strengthened in higher GDP *per capita* countries, while the negative effect of conspiracy beliefs is intensified in lower GDP *per capita* countries.

*Hypothesis 2*: The Gini index moderates the effect of satisfaction with the HCS (H2a), trust in political institutions (H2b) and conspiracy theory beliefs (H2c) on vaccination intention. The positive impacts of political trust and satisfaction with the HCS are stronger in countries with lower Gini index, while the negative effect of conspiracy theory beliefs is exacerbated in countries with a high Gini index.

### Perceived corruption

Prior research has highlighted the significant effects of corruption levels on COVID-19 vaccination.^14^ More specifically, countries with higher perceived pre-COVID-19 public sector corruption were less successful in achieving sufficient vaccination levels during the pandemic. Farzanegan and Hofmann^14^ argue that corruption undermines the country’s ability to implement public policies that are critical for undertaking large-scale health programmes, such as mass immunization. Additionally, studies show a relationship between conspiracy theories and corruption,^5^ with conspiracy theories being more common in countries where institutions are seen as untrustworthy or with a high perception of public sector corruption.^15^ Studies also show that corruption lowers trust in political institutions.^16^

The present study aims to explore whether perceived corruption moderates the relationship between conspiracy theories, trust in political institutions, satisfaction with the HCS, and COVID-19 vaccination intention. We argue that in high-corruption societies, conspiracy theories can more substantially undermine vaccination intentions since they may provide an individual with an increased sense of control and explain situations that are unfair or manipulated because of corruption. Similarly, high perceptions of corruption may undermine trust in political institutions, reducing adherence to vaccination policies. In addition, corruption may fuel dissatisfaction with the HCS,^17^ owing to perceived injustice or mismanagement. In sum, in countries with higher perceived corruption, there is a lack of trust in public institutions and HCSs. This pervasive scepticism could amplify the effect of deleterious beliefs on vaccination intention.

*Hypothesis 3:* The perceived corruption index moderates the effect of satisfaction with the HCS (H3a), trust in political institutions (H3b), and conspiracy theory beliefs (H3c) on vaccination intent. The positive effects of political trust and satisfaction with the HCS are stronger in countries with lower perceived corruption, while the negative impact of conspiracy theory beliefs is stronger in countries with a high perceived corruption index.

### Index of individualism and collectivism

The individualism and collectivism index measures how independent individuals feel in society and how connected members of the society are.^18^ Individualism does not refer to egoism but rather to the extent to which society expects individuals to make individual choices and decisions based on their unique needs. In individualistic societies loosely connected individuals view themselves as independent actors. Collectivism, on the other hand, is a cultural orientation where the individual perceives his or her place in society as socially determined and has a high level of interdependence and closer interpersonal relationships with others. Collectivistic societies emphasize group cohesion and promote group goals above individual desires.^18^

Although a collectivist cultural orientation is linked to greater willingness to get vaccinated,^19^ members of collectivist societies are more likely to hold conspiracy theory beliefs,^5^ which are a predictor of vaccine hesitancy.^1^^,^^20^^,^^21^ A moderation effect might thus be plausible, whereby the relationship between conspiracy theories and COVID-19 vaccination intention may vary based on the level of the country’s collectivism. We argue that collectivistic societies may experience a stronger negative effect of conspiracy beliefs on vaccination intention because of the higher tendency of negative beliefs to spread in tightly knit communities, where individuals may have a stronger reliance on shared values, and collective decision-making.

*Hypothesis 4*: The individualism/collectivism factor moderates the effect of beliefs in conspiracy theories on vaccination intention, intensifying the negative relationship in more collectivist societies.

*Research question 1*: Does the individualism/collectivism index moderate the relationship between trust in political institutions (RV1a), satisfaction with the HCS (RV1b) and intention to vaccinate?

### Power distance

According to Hofstede,^18^ power distance index (PDI) reflects the extent to which members of a society accept and expect an unequal distribution of power. Societies with high PDI scores are more likely to accept a hierarchical order and an unequal distribution of power in which subordinates expect to be told what to do. In contrast, societies with low PDI scores emphasize egalitarianism, expect power relations to be more democratic and encourage subordinates to challenge superiors when justified.

A comparison of the USA and China found that the public in China, where there is also a higher perceived power distance, is more supportive of conspiracy theories.^22^ Hornsey and Pearson^5^ reported the link between higher PDI and conspiracy theories at both the individual and macro levels. In addition, Kaasa and Andriani^23^ show that individual distrust in institutions is lower where PDI is lower. The authors hypothesize that lower PDI may induce individuals to feel that institutions are not responsive to citizens’ concerns.

In higher PDI societies, where hierarchy and authority are emphasized, individuals often align with the views of power figures. If these figures spread misinformation or conspiracy theories, it can significantly reduce vaccination intentions. Additionally, in such societies, trust in institutions and satisfaction with the HCS might have a weaker effect on vaccination intentions, as they may be viewed as unresponsive.

*Hypothesis 5:* The power distance index moderates the effect of satisfaction with the HCS (H5a), trust in political institutions (H5b), and conspiracy theory beliefs (H5c) on vaccination intention. The positive effects of political trust and satisfaction with the HCS are stronger in low power distance countries, while the negative impact of conspiracy theory beliefs is exacerbated in countries with a high-power distance index.

## Methods

The study used the European Social Survey 10 database,^24^ which includes a specific module designed to study the attitudes and behaviours of Europeans towards the COVID-19 pandemic. The data collection period for this study spanned from September 2020 to September 2022. Thirty-one countries were included in the tenth wave. The countries included in the present analysis were selected based on their meeting two entry criteria: (i) those that include the COVID-19 module (as this was optional) and (ii) countries for which all the macro data were available. Based on the criteria, our final sample consisted of 26 countries: Austria, Belgium, Bulgaria, Switzerland, Germany, Spain, Finland, United Kingdom, Greece, Croatia, Hungary, Ireland, Israel, Iceland, Italy, Lithuania, Latvia, North Macedonia, Netherlands, Norway, Poland, Portugal, Serbia, Sweden, Slovenia and Slovakia. The sample size ranged from 903 (Iceland) to 8725 (Germany) respondents.

Macro data were drawn from the World Bank,^25^^,^^26^ where we obtained data on GDP *per capita* and the Gini index, the index of collectivism and individualism, and the power distance were obtained from Hofstede’s Insights,^27^ and the index of perceived corruption was obtained from Transparency International.^28^ GDP *per capita* for all countries is provided for 2021, while the latest available Gini index varies; 2018 data were used for Slovakia, Poland, Norway, North Macedonia and Germany, 2017 for Iceland, and 2020 for the remaining countries.

### Measures

#### Within-level variables (level-1 variables)

Satisfaction with the country’s HCS was evaluated by asking, ‘What do you think about the state of healthcare in your country today?’ Participants answered on an 11-point scale (0 = extremely poor; 10 = extremely good).

Trust in political institutions comprises three indicators: trust in parliament, in politicians and in political parties. All indicators were measured on an 11-point scale (0 = not at all trusting; 10 = completely trusting) (Cronbach’s α = 0.91).

Conspiracy theories were measured with three indicators on a 5-point Likert scale (1 = strongly agree; 5 = strongly disagree). The first conspiracy theory was ‘A small secret group of people are responsible for making all the important decisions in world politics’; the second was related to scientists ‘Groups of scientists manipulate, fabricate or cover up evidence to mislead the public’, and the third was related to COVID-19, namely, “The coronavirus is the result of a deliberate and covert effort by a government or organization’. The values were reverse coded so that a higher value represents a higher endorsement of conspiracy beliefs (Cronbach’s α = 0.79).

#### Between-level variables (level-2 variables)

GDP was measured as GDP *per capita* in a given year in US dollar currency.^25^ The Gini coefficient of income inequality was measured on a scale from 0 to 100, with higher proportions indicating greater inequality.^26^ A country’s individualism score was tapped on a scale from 0 to 100, with higher values indicating an individualistic orientation and lower values indicating a collectivistic orientation. Power distance was also measured on a scale of 0–100, with higher values indicating greater distance.^27^ The Perceived Corruption Index, ranging from 0 to 100, measures the perception of public sector corruption, with 0 indicating a high perception of corruption.^28^

#### Outcome variable

Intention to be vaccinated against COVID-19 was measured by the following question: Will you be vaccinated against coronavirus with a vaccine approved by the national regulatory authority in your country? The possible answers were (1) Yes, I will be vaccinated, (2) Yes, I have already been vaccinated and (3) No. Since we were interested mainly in those who have accepted or would accept getting vaccinated (i.e. vaccination intention expressed for a past and future event), in comparison to those who would not, we recoded the values into a dummy variable (0 = No, 1 = Yes). Value 1 represents those who already were vaccinated at the time of the survey and those who were planning to get vaccinated. Notably, 85.7% of the respondents were categorized as 1, indicating a high level of vaccination intention in the sample.

### Statistical analyses

Multilevel models were employed to analyze relationships between individuals and their socioenvironmental contexts, recognizing that individuals or social groups are nested within these hierarchical structures.^29^ To explore determinants of COVID-19 vaccination in various countries and their variation based on societal macro characteristics, we utilized a two-level moderation regression model with random intercepts and random slopes, allowing for an interaction between the two levels.^30^

In the analysis, no fewer than ten level 2 units were included as per prior studies.^31^^,^^32^ We addressed potential bias from missing data, a common issue in multilevel analysis,^29^ by conducting Little’s test for MCAR,^33^ and identified that 18 countries violated this assumption. Therefore, we used the full information maximum likelihood estimator in the multilevel analysis,^29^ to manage incomplete data and ensure unbiased parameter estimates and accurate standard errors.^34^

Mplus 8.3 was used for the multilevel analysis. The analysis was performed in several steps using a maximum likelihood estimator with robust standard errors. Model fit was evaluated using log-likelihood (Log-L), Akaike’s information criterion and the Bayesian information criterion. First, a null model without predictors was estimated to provide a baseline model. The threshold in this model represents the log-odds of the probability of vaccination intention when all predictors are at their reference levels. We then added level-1 predictors with random intercepts and fixed slopes (Model 1), which allowed the vaccination intention to vary across countries, while assuming that the effects of predictors remain consistent across all countries. Level-1 predictors were group-mean centred to control for within-group effects and to allow the interpretation of coefficients as the effect of the predictor within groups.^35^ In Model 2, level-1 predictors with random intercepts and random slopes were included, allowing the effects of individual-level predictors to vary across countries. In addition, level-2 predictors were included in the Model 3. These predictors were grand-mean centred to interpret the coefficients as the predictor’s effect across groups.^35^ Finally, cross-level interaction models were estimated to examine whether the level-2 predictors moderated the relationships at level-1, while marginal effects were calculated for a clearer understanding of the interactions.

In the cross-level interaction models, we applied the Benjamini–Hochberg False Discovery Rate correction to control for multiple comparisons to ensure robust findings. To mitigate the impact of unequal outcome variable distribution, we conducted a repeated analysis on a subsample balanced by vaccination status.

## Results

Basic descriptive statistics (*M*, SD) and bivariate correlations between all variables included in the multilevel analysis are shown in Supplementary table S1.

Table 1 displays the multilevel analysis. Model 0, the null model, forms the baseline. Incorporating individual-level predictors into Model 1 significantly improved model fit. Vaccination intention was significantly predicted by satisfaction with the HCS (*b *=* *0.048, *P *<* *0.001), conspiracy beliefs (*b *=* −*0.697, *P* < 0.001), and trust in political institutions (*b *=* *0.138, *P *<* *0.001). Higher satisfaction with the HCS and political trust increased vaccination likelihood, while conspiracy beliefs reduced it. Random slopes of Model 2 revealed varying relationships between level-1 predictors and vaccination intent across countries, underlining the relevance of societal context. Adding level-2 predictors did not significantly predict vaccination intent, possibly owing to the lower statistical power with only 26 countries included in the analysis. Our focus, however, was not on the direct effects of level-1 and level-2 variables on vaccination intention but on level-2 variables’ moderating effect.

**Table 1:** Multilevel models.

	Model 0 (null model)			Model 1 (fixed slopes)			Model 2 (random slopes)			Model 3 (level-2 predictors)		
	Estimate (SE)	*z*	*P*	Estimate (SE)	*z*	*P*	Estimate (SE)	*z*	*P*	Estimate (SE)	*z*	*P*
Level-1 predictors												
Satisfaction with HCS				0.048 (0.011)	4.294	0.000	0.064 (0.011)	5.784	0.000	0.062 (0.012)	5.355	0.000
Trust in PI				0.138 (0.023)	5.922	0.000	0.120 (0.012)	9.738	0.000	0.121 (0.014)	8.467	0.000
Conspiracy beliefs				−0.697 (0.049)	−14.297	0.000	−0.739 (0.050)	−14.907	0.000	−0.729 (0.049)	−14.848	0.000
Level-2 predictors												
GDP										−0.003 (0.007)	−0.354	0.724
GINI										−0.004 (0.011)	−0.372	0.710
CIND										0.005 (0.008)	0.640	0.522
IDV										0.002 (0.005)	0.375	0.708
PDI										−0.001 (0.002)	−0.603	0.546
Treshold	−1.974 (0.212)	−9.313	0.000	−2.287 (0.060)	−37.996	0.000	−2.014 (0.029)	−70.201	0.000	−1.974 (0.375)	−5.269	0.000
Variance_σ2_				0.934 (0.042)	22.356	0.000	1.322 (0.023)	58.545	0.000	0.970 (0.099)	9.853	0.000
Model fit												
Log-L	−16742.933			−13364.373			−13287.917			−13283.778		
AIC	33489.865			26738.745			26591.835			26593.556		
BIC	33507.436			26766.195			26635.755			26706.240		

Note: Satisfaction with HCS, satisfaction with the healthcare system; Trust in PI, trust in political institutions; GDP, GDP *per capita*; GINI, Gini index; CIND, perceived corruption index; IDV, individualism/collectivism index; PDI, power distance index, Log-L, Log likelihood value; AIC, Akaike’s information criterion, BIC, Bayesian information criterion.

**Figure 1:**
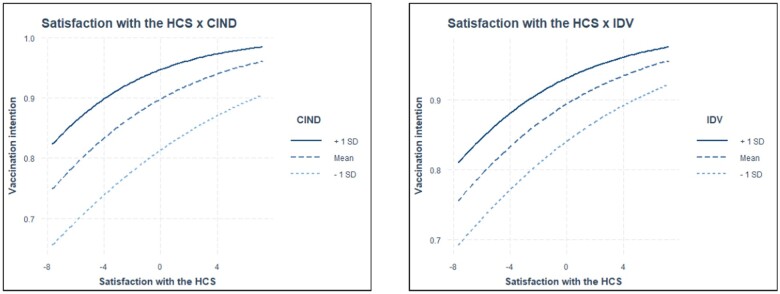
Moderating effect of perceived corruption (CIND) and individualism/collectivism (IDV) on satisfaction with the healthcare system and vaccination.

**Figure 2:**
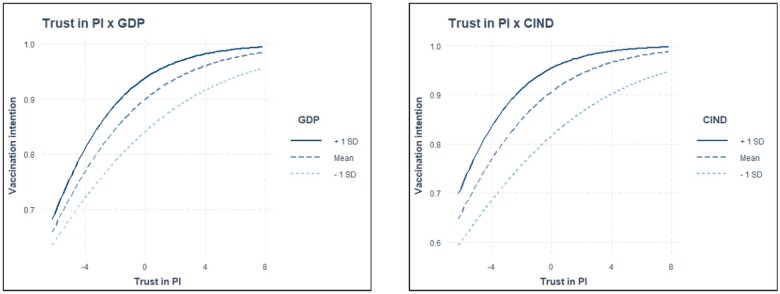
Moderating effect of GDP and perceived corruption (CIND) on trust in political institutions and vaccination.

**Figure 3:**
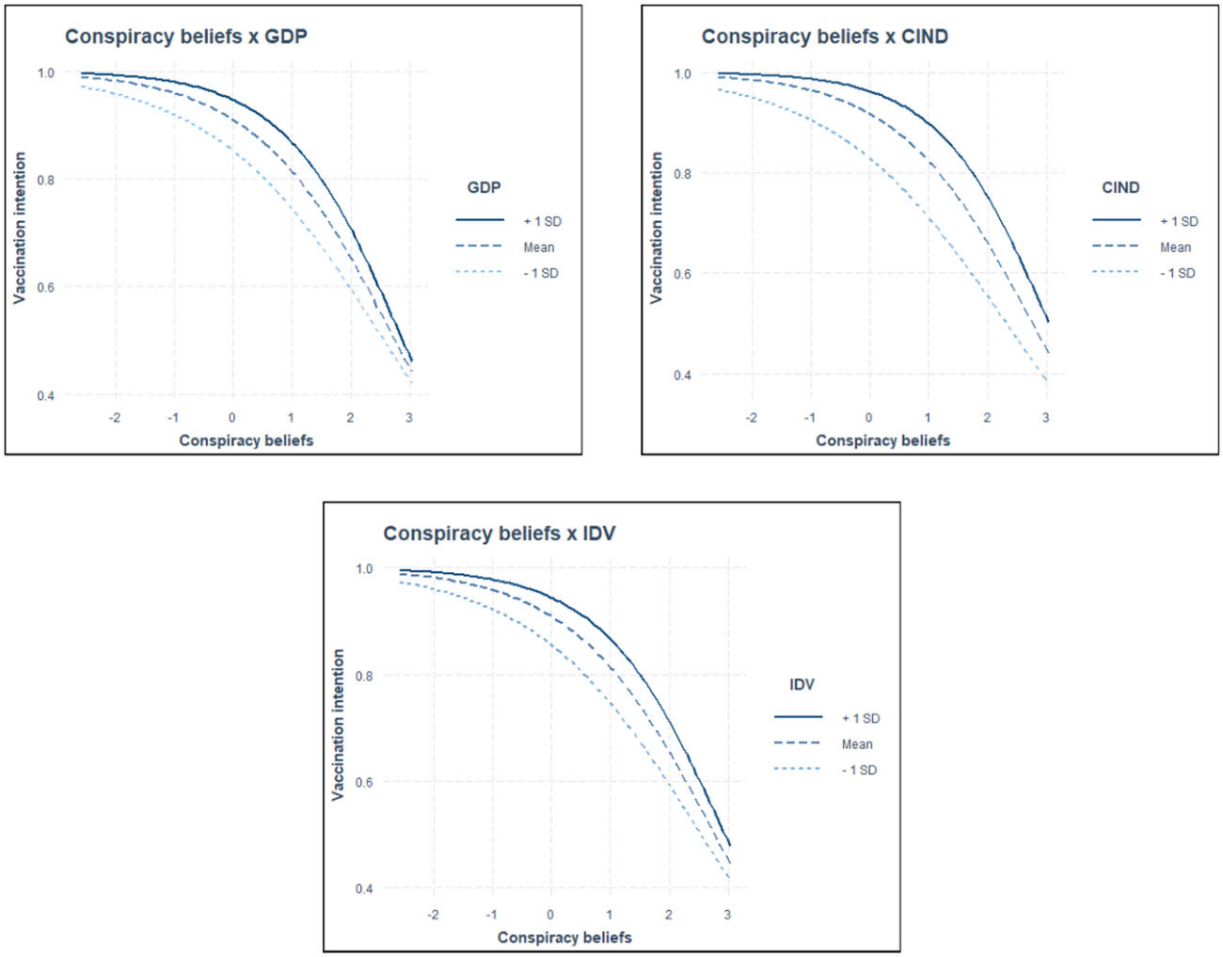
Moderating effect of GDP, perceived corruption (CIND) and individualism/collectivism (IDV) on trust in political institutions and vaccination.

The analysis revealed several statistically significant cross-level interactions (Supplementary table S2). The positive effect of satisfaction with the HCS on vaccination intention was stronger in countries with lower perceived corruption (*γ* = 0.003, *P *<* *0.01), and more individualistic countries (*γ* = 0.001, *P *<* *0.05), confirming hypothesis H3a. In addition, the positive effect of trust in political institutions on vaccination uptake was stronger in countries with higher GDP *per capita* (γ = 0.003, *P *<* *0.05), and in countries with lower perceived corruption (γ = 0.005, *P *<* *0.001), supporting hypotheses H1b and H3b. Finally, the negative effect of conspiracy beliefs on vaccine uptake was stronger in countries with lower GDP *per capita* (γ= −0.008, *P *<* *0.05), with higher corruption perception (*γ* = −0.013, *P* < 0.001), and in more collectivistic countries (*γ* = −0.008, *P *<* *0.01), corroborating hypotheses H1c, H3c and H4. Other hypotheses were not supported due to non-significant cross-level interactions. The marginal effects (estimated marginal means) (Supplementary tables S3–S5) show significant but small moderating effects, likely influenced by the uneven distribution of vaccination intention. A supplementary analysis on a balanced subsample reveals more pronounced effects (see Supplementary material). For a visual representation of statistically significant interactions, see figures 1–3.

## Discussion

This study’s results highlight that in countries with low perceived corruption and higher individualism, satisfaction with the HCS has a greater positive effect on COVID-19 vaccination intent. Trust in political institutions affects vaccination intent more positively in higher GDP *per capita* and less corrupt countries. These results underscore the variability of the effects of political trust and satisfaction with the HCS on vaccination intent across diverse economic and cultural contexts.

Previous research suggests that higher GDP *per capita* may positively affect institutional trust.^36^ Thus, in such countries the effect of trust in political institutions on vaccination intentions is stronger, as indicated by our results. Similarly, lower perceived corruption levels increase trust in economic and political institutions and civil society.^37^ Consequently, in societies with less perceived corruption, the positive effect of satisfaction with the HCS and trust in political institutions on vaccination intention is stronger because corruption does not undermine trust and satisfaction.

Regarding the cultural aspects, the individualistic cultural context strengthens the positive effect of satisfaction with the HCS on COVID-19 vaccination intention. In the context of cultural orientations, it is important to consider the scope of trust fostered by them. Individualistic societies often cultivate a generalized form of trust that extends beyond in-group boundaries,^38^ which is why the effects of satisfaction on vaccination intention strengthen in such contexts. Conversely, collectivist cultures tend to develop trust within tightly knit in-groups, potentially limiting the extent of trust placed in wider institutions and unfamiliar individuals.^38^ This means that they may inadvertently foster an environment more conducive to dissatisfaction towards broader institutions.

Moreover, we found that conspiracy beliefs had a stronger negative effect on vaccination intention in countries characterized by lower levels of economic development, higher perceived corruption and collectivism. In less economically developed countries, limited access to health-related information can amplify the adverse effects of conspiracy theory beliefs on vaccination intention. In addition, in collectivistic societies, the close-knit nature may strengthen the negative effect of conspiracy beliefs on vaccination intentions.

In summary, our study shows that economic and cultural contexts moderate the association between satisfaction with the HCS, trust in political institutions, conspiracy beliefs and COVID-19 vaccination intention. These findings reveal the complexity of vaccine attitudes and decision-making, highlighting that vaccination decisions are shaped by both individual choices and wider societal characteristics. In societies characterized by lower corruption and higher individualism, the positive effect of satisfaction with HCS strengthens. Additionally, the positive effect of trust in political institutions on vaccination strengthens in countries with higher GDP *per capita* and lower perceived corruption. In contrast, in societies that are less economically developed, perceived as more corrupt and more collectivistically oriented, conspiracy beliefs tend to have a stronger negative effect on vaccination intention. These findings highlight the need for context-specific approaches when promoting vaccination and provide a basis for more detailed research on vaccination intention in different social contexts. To effectively address conspiracy beliefs and political distrust, interventions should consider both the individual and macro level.^39^ Misinformation requires special attention in vaccine promotion.^39^ Roozenbeek et al.^40^ highlight the importance of systemic-level interventions in countering misinformation, including geopolitical actions and legislative measures. At the same time, Kozyreva et al.^39^ offer interventions aimed at the individual level, including nudging, refutation strategies and boosts.

### Limitations of the study

The present study highlighted the role of contextual factors in understanding the intention to get vaccinated against COVID-19; however, several limitations must be addressed. Firstly, the study is cross-sectional; therefore, causal inferences cannot be made. Secondly, the estimates of cross-level interaction effects are relatively small, which warrants careful interpretation of the results. Moreover, the number of countries was only 26; among those, the majority were EU countries, which may limit the generalizability of the results to non-European cultural contexts. In addition, the outcome variable (vaccination intention) was a categorical variable. If this construct or similar constructs such as vaccine hesitancy were used and measured with multiple items, we could assess greater variability in vaccine intent and attitudes. Next, the study addressed several complex relationships which are currently under-researched; however, the explanatory mechanisms of these relationships were not empirically evaluated. Future research should thus closely examine the nature of these relationships, to provide a more comprehensive understanding of vaccination intention and how wider socioeconomic and cultural environmental characteristics contribute to determinants of vaccine behaviour.

## Supplementary Material

ckae022_Supplementary_Data

## Data Availability

The dataset is available in the ESS (European Social Survey) repository, accessible via the following link: https://ess.sikt.no/en/datafile/f37d014a-6958-42d4-b03b-17c29e481d3d/256?tab=documentation. Key points The negative effect of conspiracy beliefs on COVID-19 vaccination is stronger in countries with high corruption, collectivist countries, and in countries with lower GDP *per capita*. Trust in politics boosts the intention to get vaccinated more in economically developed and less in corrupt countries. Satisfaction with the healthcare system effects vaccination more positively in countries that are less corrupt and more individualistic. Public health policies should tailor communication strategies to cultural and economic contexts, addressing conspiracy beliefs and strengthening healthcare satisfaction to improve vaccination rates.
