# Supplementary material for: Multilevel analysis of COVID-19 vaccination intention: the moderating role of economic and cultural country characteristics
Source: Eur J Public Health. 2024 Feb 20;34(2):380–6. doi: 10.1093/eurpub/ckae022 (PMC10990524; doi:10.1093/eurpub/ckae022)
Supplement: ckae022_Supplementary_Data [file ckae022_supplementary_data.zip › ckae022_Supplementary_Data/ejph-2023-07-om-0413-File006.pdf]

Table S1. Descriptive statistics

	M	*SD*	1.	2.	3.	4.	5.	6.	7.	8.
1. COVID-19 vaccination	/	/	-							
2. Satisfaction with HCS	5.38	2.70	0.18 (p = 0.01)	-						
3. Trust in PI	3.80	2.38	0.22 (p = 0.01)	0.46 (p = 0.01)	-					
4. Conspiracy beliefs	2.61	1.03	-0.32 (p = 0.01)	-0.27 (p = 0.01)	-0.37 (p = 0.01)	-				
5. GDP	48985	17949	0.19 (p = 0.01)	0.26 (p = 0.01)	0.31 (p = 0.01)	-.032 (p = 0.01)	-			
6. GINI	31.86	4.06	-0.14 (p = 0.01)	-0.13 (p = 0.01)	-0.22 (p = 0.01)	0.16 (p = 0.01)	-0.40 (p = 0.01)	-		
7. CIND	65.04	15.51	0.25 (p = 0.01)	0.32 (p = 0.01)	0.33 (p = 0.01)	-0.41 (p = 0.01)	0.80 (p = 0.01)	-0.41 (p = 0.01)	-	
8. IDV	60.32	16.92	0.21 (p = 0.01)	0.17 (p = 0.01)	0.28 (p = 0.01)	-0.26 (p = 0.01)	0.45 (p = 0.01)	-0.31 (p = 0.01)	0.46 (p = 0.01)	-
9. PDI	42.66	20.52	-0.19 (p = 0.01)	-0.20 (p = 0.01)	-0.15 (p = 0.01)	0.24 (p = 0.01)	-0.41 (p = 0.01)	-0.06 (p = 0.01)	-0.38 (p = 0.01)	-0.54 (p = 0.01)

Note. Satisfaction with HCS = satisfaction with the healthcare system, Trust in PI = trust in political institutions, GDP = GDP *per capita*, GINI = Gini index, CIND = perceived corruption index, IDV = individualism/collectivism index, PDI = power distance index.

Table S2. Cross-level interactions

	Estimate (SE)	z	p	Adjusted p
Satisfaction with HCS × GDP	0.001 (0.001)	1.274	0.203	0.234
Satisfaction with HCS × GINI	-0.003 (0.003)	-1.071	0.284	0.284
Satisfaction with HCS × CIND	0.003 (0.001)	3.297	0.001	0.004
Satisfaction with HCS × IDV	0.001 (0.001)	2.365	0.018	0.038
Satisfaction with HCS × PDI	-0.001 (0.000)	-2.130	0.033	0.056
Trust in PI × GDP	0.003 (0.001)	2.730	0.006	0.018
Trust in PI × GINI	-0.005 (0.004)	-1.208	0.227	0.243
Trust in PI × CIND	0.005 (0.001)	6.943	0.000	0.000
Trust in PI × IDV	0.002 (0.001)	1.958	0.050	0.075
Trust in PI × PDI	-0.001 (0.001)	-1.763	0.078	0.106
Conspiracy beliefs × GDP	-0.008 (0.003)	-2.601	0.009	0.023
Conspiracy beliefs × GINI	0.022 (0.011)	2.118	0.034	0.057
Conspiracy beliefs × CIND	-0.013 (0.002)	-6.806	0.000	0.000
Conspiracy beliefs × IDV	-0.008 (0.002)	-3.212	0.001	0.004
Conspiracy beliefs × PDI	0.003 (0.002)	1.364	0.173	0.216

Note. Adjusted p values reflect p-values that have been adjusted using the Benjamini-Hochberg False Discovery Rate (FDR) correction for multiple comparisons. Satisfaction with HCS = satisfaction with the healthcare system, Trust in PI = trust in political institutions, GDP = GDP *per capita*, GINI = Gini index, CIND = perceived corruption index, IDV = individualism/collectivism index, PDI = power distance index.

Table S3. Marginal effects of corruption, individualism/collectivism, and satisfaction with the HCS on vaccination intention

**CIND**	**Satisfaction with the HCS**	**Predicted probability of vaccination**	**95% CI**
-1SD	Low	0.60	[0.49, 0.69]
	Mean	0.81	[0.74, 0.87]
	High	0.93	[0.89, 0.95]
Mean	Low	0.68	[0.61, 0.75]
	Mean	0.90	[0.87, 0.92]
	High	0.97	[0.96, 0.98]
+1SD	Low	0.75	[0.65, 0.83]
	Mean	0.88	[0.92, 0.97]
	High	0.99	[0.98, 0.99]
**IDV**	**Satisfaction with the HCS**	**Predicted probability of vaccination**	**95% CI**
-1SD	Low	0.63	[0.52, 0.73]
	Mean	0.84	[0.77, 0.89]
	High	0.94	[0.91, 0.96]
Mean	Low	0.69	[0.61, 0.77]
	Mean	0.89	[0.86, 0.92]
	High	0.97	[0.96, 0.98]
+1SD	Low	0.75	[0.64, 0.83]
	Mean	0.93	[0.89, 0.96]
	High	0.98	[0.97, 0.99]

Note. For parsimony, 'Low', 'Mean', and 'High' for the independent predictor represent its minimum, average, and maximum values, respectively. Satisfaction with HCS = satisfaction with the healthcare system, CIND = perceived corruption index, IDV = individualism/collectivism index.

Table S4. Marginal effects of GDP, corruption, and trust in political institutions on vaccination intention

GDP	Trust in PI	Predicted probability of vaccination	95% CI
-1SD	Low	0.56	[0.43, 0.67]
	Mean	0.84	[0.77, 0.90]
	High	0.96	[0.93, 0.97]
Mean	Low	0.55	[0.46, 0.64]
	Mean	0.90	[0.86, 0.93]
	High	0.99	[0.98, 0.99]
+1SD	Low	0.55	[0.42, 0.67]
	Mean	0.94	[0.90, 0.96]
	High	0.99	[0.99, 1.00]
**CIND**	**Trust in PI**	**Predicted probability of vaccination**	**95% CI**
-1SD	Low	0.51	[0.41, 0.62]
	Mean	0.82	[0.75, 0.87]
	High	0.95	[0.92, 0.97]
Mean	Low	0.53	[0.45, 0.61]
	Mean	0.91	[0.88, 0.93]
	High	0.99	[0.98, 0.99]
+1SD	Low	0.55	[0.43, 0.67]
	Mean	0.95	[0.93, 0.97]
	High	1.00	[1.00, 1.00]

Note. For parsimony, 'Low', 'Mean', and 'High' for the independent predictor represent its minimum, average, and maximum values, respectively. Trust in PI = trust in political institutions, GDP = GDP *per capita*, CIND = perceived corruption.

Table S5. Marginal effects of GDP, corruption, individualism/collectivism, and conspiracy beliefs on vaccination intention

**GDP**	**Conspiracy beliefs**	**Predicted probability**	**95% CI**
-1SD	Low	0.98	[0.96, 0.99]
	Mean	0.85	[0.78, 0.90]
	High	0.43	[0.31, 0.55]
Mean	Low	0.99	[0.99, 0.99]
	Mean	0.91	[0.88, 0.94]
	High	0.45	[0.36, 0.54]
+1SD	Low	1.00	[1.00, 1.00]
	Mean	0.91	[0.91, 0.97]
	High	0.47	[0.34, 0.60]
**CIND**	**Conspiracy beliefs**	**Predicted probability**	**95% CI**
-1SD	Low	0.97	[0.96, 0.98]
	Mean	0.38	[0.76, 0.88]
	High	0.39	[0.29, 0.49]
Mean	Low	0.99	[0.99, 1.00]
	Mean	0.92	[0.89, 0.94]
	High	0.45	[0.37, 0.53]
+1SD	Low	1.00	[1.00, 1.00]
	Mean	0.96	[0.94, 0.98]
	High	0.51	[0.39, 0.63]
**IDV**	**Conspiracy beliefs**	**Predicted probability**	**95% CI**
-1SD	Low	0.98	[0.97, 0.99]
	Mean	0.86	[0.79, 0.90]
	High	0.42	[0.31, 0.54]
Mean	Low	0.99	[0.99, 0.99]
	Mean	0.91	[0.87, 0.94]
	High	0.45	[0.36, 0.55]
+1SD	Low	1.00	[0.99, 1.00]
	Mean	0.94	[0.91, 0.97]
	High	0.49	[0.36, 0.61]

Note. For parsimony, 'Low', 'Mean', and 'High' for the independent predictor represent its minimum, average, and maximum values, respectively. GDP = GDP *per capita*, CIND = perceived corruption index, IDV = individualism/collectivism index.
