# Supplementary material for: Multilevel analysis of COVID-19 vaccination intention: the moderating role of economic and cultural country characteristics
Source: Eur J Public Health. 2024 Feb 20;34(2):380–6. doi: 10.1093/eurpub/ckae022 (PMC10990524; doi:10.1093/eurpub/ckae022)
Supplement: ckae022_Supplementary_Data [file ckae022_supplementary_data.zip › ckae022_Supplementary_Data/ejph-2023-07-om-0413-File007.pdf]

Supplementary Materials: Subsample Analyses

To account for the unequal distribution of the outcome variable, i.e. vaccination status, and to further examine the robustness of the results, we repeated the analyses on a subsample where the population is equally distributed with respect to the outcome variable (N = 13828). Multilevel models (Table 1) revealed the same results as the main analysis. Model fit improved with the addition of random slopes to predictors. Satisfaction with the healthcare system and trust in political institutions remained positive predictors of vaccination intention, while greater belief in conspiracies decreased the likelihood of vaccination intention. In Model 4, perceived corruption emerged as a significant predictor of vaccination intention; specifically, lower perceived corruption increased vaccination intention (in the main model, all level-2 predictors were statistically insignificant). Cross-level interactions, which were analyzed in the main results following the Benjamini-Hochberg FDR correction, are presented in Table 2. All results remained consistent with the main findings.

Table 1. Multilevel models

	Model 0 (null model)			Model 1 (fixed slopes)			Model 2 (random slopes)			Model 3 (level-2 predictors)		
	Estimate (SE)	z	p	Estimate (SE)	z	p	Estimate (SE)	z	p	Estimate (SE)	z	p
Level-1 predictors												
Satisfaction with HCS				0.057 (0.011)	5.107	0.000	0.066 (0.016)	4.085	0.000	0.062 (0.012)	5.037	0.000
Trust in PI				0.137 (0.030)	4.633	0.000	0.118 (0.015)	7.829	0.000	0.123 (0.018)	6.867	0.000
Conspiracy beliefs				-0.729 (0.051)	-14.174	0.000	-0.736 (0.055)	-13.355	0.000	-0.735 (0.053)	-13.756	0.000
Level-2 predictors												
GDP										-0.019 (0.015)	-1.264	0.206
GINI										0.017 (0.046)	0.369	0.712
CIND										0.062 (0.009)	6.972	0.000
IDV										0.012 (0.029)	0.436	0.663
PDI										-0.006 (0.004)	-1.448	0.148
Treshold $_{\beta 0}$	0.000 (0.255)	0.000	1.000	-0.621 (0.068)	-9.196	0.000	-0.312 (0.046)	-6.811	0.000	-0.609 (0.150)	-4.059	0.000
Variance $_{\sigma 2}$				0.822 (0.044)	18.839	0.000	1.375 (0.144)	9.566	0.000	0.490 (0.136)	3.587	0.000
Model fit												
Log-L	-9584.8			-6263.5			-6226.2			-6209.7		
AIC	19171.7			12536.9			12468.3			12445.4		
BIC	19179.2			12573.9			12527.6			12500.4		

Note. Satisfaction with HCS = satisfaction with the healthcare system, Trust in PI = trust in political institutions, GDP = GDP *per capita*, GINI = Gini index, CIND = perceived corruption index, IDV = individualism/collectivism index, PDI = power distance index.

Table 2. Cross-level interactions

	Estimate (SE)	z	p	Adjusted p
Satisfaction with HCS × CIND	0.002 (0.000)	3.545	0.000	0.000
Satisfaction with HCS × IDV	0.001 (0.000)	2.873	0.007	0.011
Trust in PI × GDP	0.004 (0.000)	7.644	0.000	0.000
Trust in PI × CIND	0.006 (0.000)	10.463	0.000	0.000
Conspiracy beliefs × GDP	-0.008 (0.001)	-5.705	0.000	0.000
Conspiracy beliefs × CIND	-0.012 (0.002)	-8.134	0.000	0.000
Conspiracy beliefs × IDV	-0.006 (0.001)	-4.720	0.000	0.000

Note. In the subsample analysis, only interactions that were significant after applying the Benjamini-Hochberg FDR correction in the main sample were included. Satisfaction with HCS = satisfaction with the healthcare system, Trust in PI = trust in political institutions, GDP = GDP *per capita*, CIND = perceived corruption index, IDV = individualism/collectivism index.

Figures 1, 2, and 3 visually present the interactions. The analysis confirms that lower perceived corruption strengthens the positive effect of satisfaction with the HCS on vaccination intention. The marginal effects presented in Table 5 provide nuanced insights into these moderated effects. Notably, in contexts of higher perceived corruption, the positive effect of satisfaction with the HCS is less pronounced. For example, while high satisfaction increases vaccination probability to 0.64 (compared to 0.17 at low satisfaction), this increase is less substantial than in settings with lower perceived corruption, where probabilities for vaccination rise from 0.32 for low satisfaction to 0.93 for high satisfaction. Similarly, cultural orientation effects this relationship: in more individualistic settings, satisfaction has a stronger positive effect on vaccination intention (0.91) than in collectivistic settings (0.71).

Figure 1. Moderating effect of perceived corruption (CIND) and individualism/collectivism (IDV) on satisfaction with the healthcare system and vaccination.

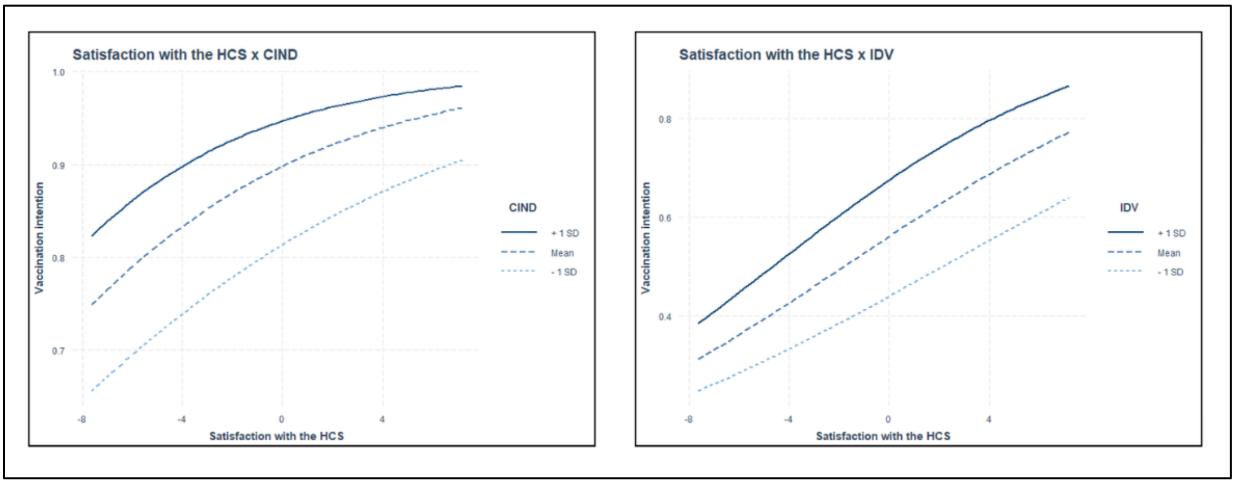

Table 5. Marginal effects of corruption, individualism/collectivism, and satisfaction with the HCS on vaccination intention.

**CIND**	**Satisfaction with the HCS**	**Predicted probability of vaccination**	**95% CI**
-1SD	Low	0.17	[0.11, 0.25]
	Mean	0.38	[0.27, 0.49]
	High	0.64	[0.52, 0.75]
Mean	Low	0.24	[0.18, 0.30]
	Mean	0.55	[0.48, 0.62]
	High	0.38	[0.78, 0.87]
+1SD	Low	0.32	[0.23, 0.43]
	Mean	0.72	[0.63, 0.79]
	High	0.93	[0.89, 0.95]
**IDV**	**Satisfaction with the HCS**	**Predicted probability of vaccination**	**95% CI**
-1SD	Low	0.20	[0.13, 0.31]
	Mean	0.44	[0.32, 0.57]
	High	0.71	[0.58, 0.81]
Mean	Low	0.25	[0.18, 0.33]
	Mean	0.56	[0.47, 0.64]
	High	0.83	[0.77, 0.88]
+1SD	Low	0.30	[0.21, 0.41]
	Mean	0.68	[0.57, 0.76]
	high	0.91	[0.86, 0.94]

For parsimony, 'Low', 'Mean', and 'High' for the independent predictor represent its minimum, average, and maximum values, respectively. Satisfaction with HCS = satisfaction with the healthcare system, CIND = perceived corruption index, IDV = individualism/collectivism index.

A similar pattern is observed with trust in political institutions, GDP, and corruption (Table 6). When GDP is higher, the positive effect of trust on vaccination intention is more pronounced,

reaching 0.98, compared to when GDP is lower at 0.75. Additionally, the positive effects are most pronounced when corruption is perceived as low (0.96), as opposed to high (0.64).

Figure 2. Moderating effect of GDP and perceived corruption (CIND) on trust in political institutions and vaccination.

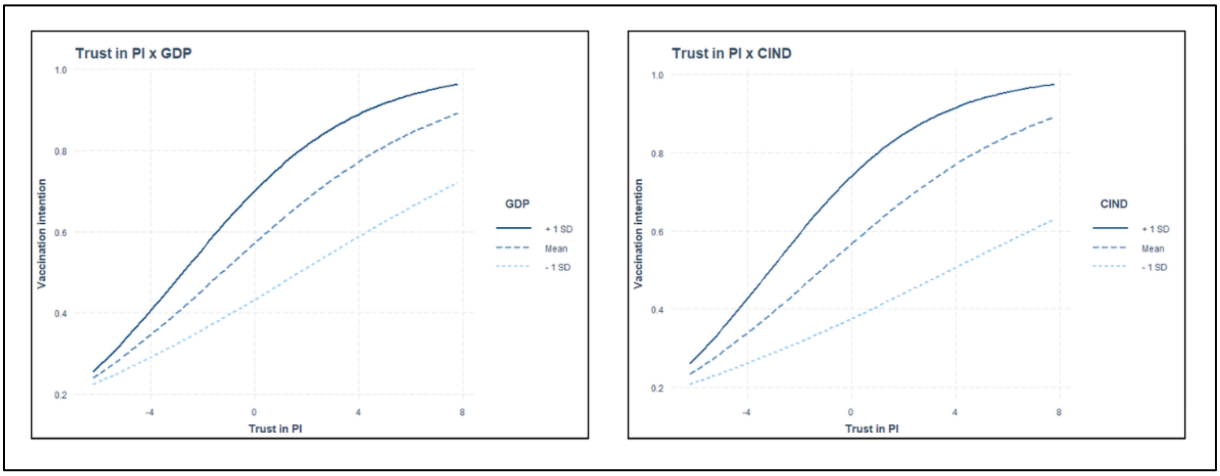

Table 6. Marginal effects of GDP, corruption and trust in political institutions on vaccination intention.

**GDP**	**Trust in PI**	**Predicted probability of vaccination**	**95% CI**
-1SD	Low	0.19	[0.12, 0.29]
	Mean	0.46	[0.33, 0.59]
	High	0.75	[0.63, 0.84]
Mean	Low	0.17	[0.13, 0.24]
	Mean	0.57	[0.49, 0.66]
	High	0.90	[0.85, 0.93]
+1SD	Low	0.16	[0.10, 0.24]
	Mean	0.69	[0.58, 0.77]
	High	0.96	[0.94, 0.98]
**CIND**	**Trust in PI**	**Predicted probability of vaccination**	**95% CI**
-1SD	Low	0.17	[0.11, 0.26]
	Mean	0.38	[0.27, 0.49]
	High	0.64	[0.51, 0.75]
Mean	Low	0.17	[0.13, 0.22]
	Mean	0.57	[0.49, 0.64]
	High	0.90	[0.86, 0.92]
+1SD	Low	0.16	[0.11, 0.23]
	Mean	0.74	[0.66, 0.81]
	High	0.98	[0.96, 0.99]

Note. For parsimony, 'Low', 'Mean', and 'High' for the independent predictor represent its minimum, average, and maximum values, respectively. Trust in PI = trust in political institutions, GDP = GDP *per capita*, CIND = perceived corruption.

Marginal effects shown in Table 7 demonstrate that in countries with lower GDP the probability of vaccination intention is 0.88 for individuals with low conspiracy beliefs, dropping significantly to 0.10 with those with high conspiracy beliefs. Additionally, when perceived corruption is high, the vaccination probability is 0.81 with low conspiracy beliefs, compared to only 0.09 with high conspiracy beliefs. In contrast, when corruption is perceived lower probabilities for vaccination are 0.14 when conspiracies are high, and 0.99 when they are low. The moderating role of individualism/collectivism demonstrates further reveals that when individualism is higher, the probability for vaccination is 0.13 when conspiracies are high, and 0.98 when they are low; contrary to when the societies are more collectivistic, when the probability for vaccination is 0.09 when beliefs are high and 0.88 when they are lower.

Figure 3. Moderating effect of GDP, perceived corruption (CIND) and individualism/collectivism (IDV) on trust in political institutions and vaccination.

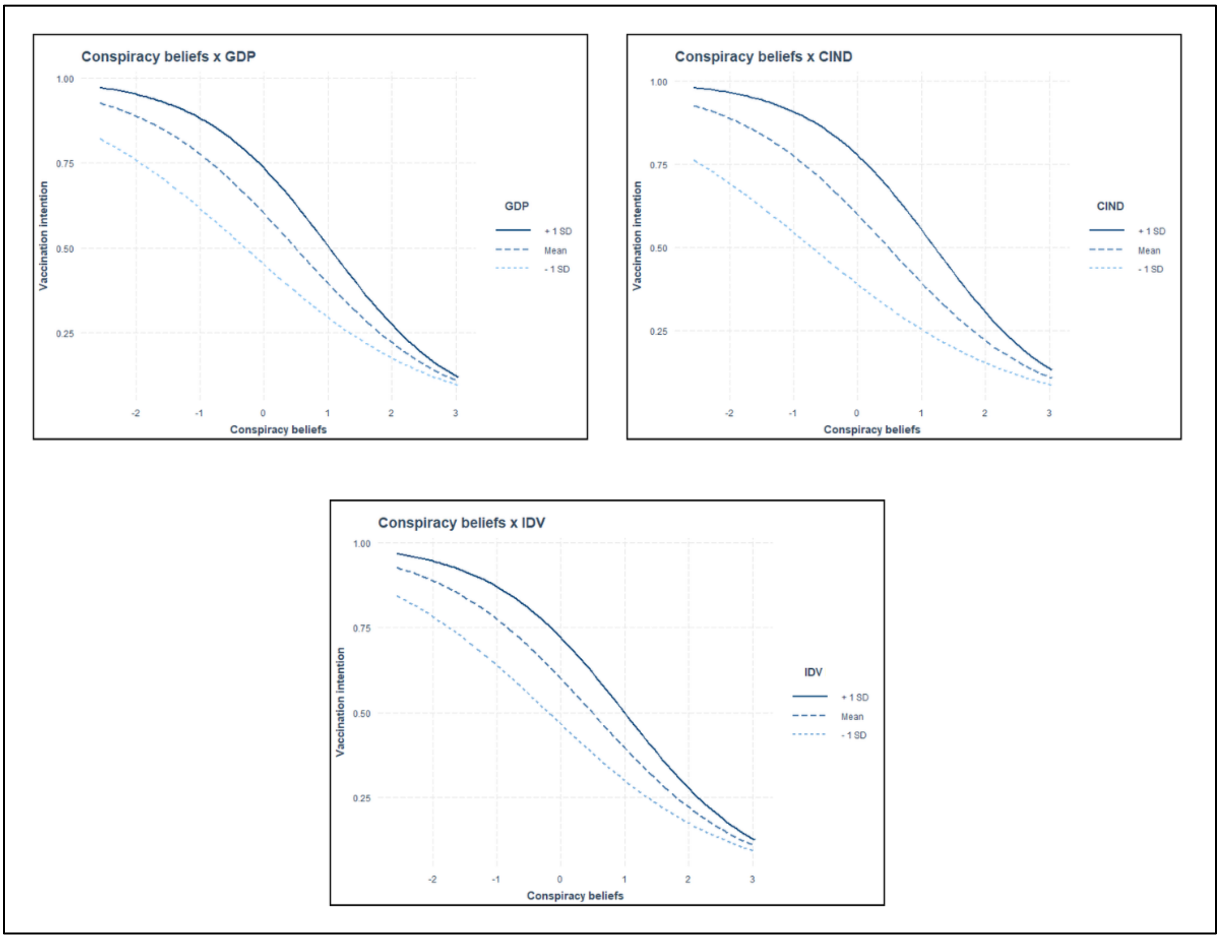

Table 7. Marginal effects of GDP, corruption, individualism/collectivism, and conspiracy beliefs on vaccination intention.

GDP	Conspiracy beliefs	Predicted probability	95% CI
-1SD	Low	0.88	[0.80, 0.93]
	Mean	0.47	[0.34, 0.61]
	High	0.10	[0.06, 0.17]
Mean	Low	0.95	[0.92, 0.97]
	Mean	0.60	[0.51, 0.69]
	High	0.11	[0.08, 0.16]
+1SD	Low	0.98	[0.97, 0.99]
	Mean	0.72	[0.61, 0.81]
	High	0.12	[0.08, 0.19]
**CIND**	**Conspiracy beliefs**	**Predicted probability**	**95% CI**
-1SD	Low	0.81	[0.71, 0.88]
	Mean	0.39	[0.28, 0.51]
	High	0.09	[0.05, 0.14]
Mean	Low	0.95	[0.93, 0.96]
	Mean	0.60	[0.52, 0.67]
	High	0.11	[0.08, 0.15]
+1SD	Low	0.99	[0.98, 0.99]
	Mean	0.78	[0.69, 0.84]
	High	0.14	[0.09, 0.20]
**IDV**	**Conspiracy beliefs**	**Predicted probability**	**95% CI**
-1SD	Low	0.88	[0.80, 0.93]
	Mean	0.47	[0.34, 0.60]
	High	0.09	[0.06, 0.16]
Mean	Low	0.95	[0.92, 0.96]
	Mean	0.60	[0.51, 0.69]
	High	0.11	[0.08, 0.16]
+1SD	Low	0.98	[0.96, 0.99]
	Mean	0.72	[0.62, 0.81]
	High	0.13	[0.08, 0.20]

Note. For parsimony, 'Low', 'Mean', and 'High' for the independent predictor represent its minimum, average, and maximum values, respectively. GDP = GDP *per capita*, CIND = perceived corruption index, IDV = individualism/collectivism index.
